# Moderate Exercise Inhibits Age-Related Inflammation, Liver Steatosis, Senescence, and Tumorigenesis

**DOI:** 10.4049/jimmunol.2001022

**Published:** 2021-01-13

**Authors:** Arianna Bianchi, Letizia Marchetti, Zoe Hall, Henrique Lemos, Michele Vacca, Hannah Paish, Kile Green, Bronte Elliott, Dina Tiniakos, João F. Passos, Diana Jurk, Derek A. Mann, Caroline L. Wilson

**Affiliations:** * Biosciences Institute, Newcastle University, Newcastle upon Tyne NE2 4HH, United Kingdom;; † Biomolecular Medicine, Department of Metabolism, Digestion and Reproduction, Imperial College London, London SW7 2AZ, United Kingdom;; ‡ Department of Biochemistry, University of Cambridge, Cambridge CB2 1QW, United Kingdom;; § Department of Biomedical Science, University of Sheffield, Sheffield S10 2TN, United Kingdom; and; ¶ Department of Physiology and Biomedical Engineering, Mayo Clinic, Rochester, MN 55905

## Abstract

Age-related chronic inflammation promotes cellular senescence, chronic disease, cancer, and reduced lifespan. In this study, we wanted to explore the effects of a moderate exercise regimen on inflammatory liver disease and tumorigenesis. We used an established model of spontaneous inflammaging, steatosis, and cancer (*nfkb1^−/−^* mouse) to demonstrate whether 3 mo of moderate aerobic exercise was sufficient to suppress liver disease and cancer development. Interventional exercise when applied at a relatively late disease stage was effective at reducing tissue inflammation (liver, lung, and stomach), oxidative damage, and cellular senescence, and it reversed hepatic steatosis and prevented tumor development. Underlying these benefits were transcriptional changes in enzymes driving the conversion of tryptophan to NAD^+^, this leading to increased hepatic NAD^+^ and elevated activity of the NAD^+^-dependent deacetylase sirtuin. Increased SIRT activity was correlated with enhanced deacetylation of key transcriptional regulators of inflammation and metabolism, NF-κB (p65), and PGC-1α. We propose that moderate exercise can effectively reprogram pre-established inflammatory and metabolic pathologies in aging with the benefit of prevention of disease.

## Key Points

- Moderate exercise reduces inflammaging, liver tumors, and chronic liver disease.
- Key molecular changes include lipid metabolism, oxidative damage, and senescence.
- Increased NAD^+^, sirtuin activity, and inflammatory and metabolic reprogramming occur.

## Introduction

Aging is associated with low-level sterile chronic inflammation (or parainflammation), cellular senescence, and declines in nutrient sensing and mitochondrial function. These changes mechanistically underpin susceptibility to age-related pathologies such as cardiovascular disease, type II diabetes, liver disease, and cancer (1). As proposed by Medzhitov (2), parainflammation is triggered by tissue malfunction and is an attempt to restore homeostasis, but if sustained, it promotes chronic inflammatory diseases that occur in the absence of an injury or infection. We previously reported that parainflammation promotes telomere dysfunction and is integrated into positive feedback loops that operate within stress and nutrient sensing networks implicated in the control of mitochondrial function and reactive oxygen species production (3). As a consequence, parainflammation aggravates cellular senescence, which in turn, through the senescence-associated secretory phenotype elevates inflammation to a pathological state. Noteworthy, senescence in liver cells stimulates fat accumulation (steatosis) in the absence of obesity (4). This finding may be relevant to a nonobese form of nonalcoholic fatty liver disease that makes up to half of all disease cases (5). Hence, parainflammation, cellular senescence, and perturbed metabolism (inflammaging) are intimately networked through to promote chronic disease states.

Regular exercise benefits maintenance of muscle mass and reduces susceptibility to age-related chronic disease and cancer (6, 7). Cohort studies in the elderly demonstrate exercise-associated reductions in biomarkers of inflammaging such as CRP and IL-6 (8). Lack of physical activity partially mediates negative health impacts of chronic disease (9). However, the molecular basis for the health benefits of exercise is poorly defined, as is the evidence that regular exercise is an effective intervention for suppressing the progression of chronic inflammation to disease states.

In this study, we asked if modest exercise can prevent inflammaging using the *nfkb1^−/−^* mouse, which spontaneously develops inflammation-driven premature aging as a model (10). The *nfkb1^−/−^* mouse lacks the p50 subunit of NF-κB, which classically heterodimerizes with RelA to positively regulate inflammatory gene transcription (11). Conversely, p50 homodimers combine with histone deacetylase 1 (HDAC1) to repress inflammatory gene expression (12, 13). Hence, p50 homodimers are emerging as key anti-inflammatory signaling factors. In support, a mutation in the murine *nfkb1* gene (*nfkb1^S340A^*) that selectively prevents assembly of p50 homodimers results in a hyperinflammatory response to liver damage (14). Similarly, in humans, several naturally occurring heterozygous mutations in *NFKB1* associate with autoinflammatory or hyperinflammatory phenotypes and, in some patients, chronic liver disease (15–17). Aged *nfkb1^−/−^* mice develop a variety of pathologies characteristic of unhealthy human aging including autoimmunity, loss of neuromuscular coordination, lung emphysema, loss of s.c. fat coupled with increased liver fat, decreased osteogenesis, and early mortality (10, 14, 18, 19). The model is also characterized by severe chronic hepatic and gastric disease that spontaneously progresses to cancer (14, 19). These observations all point to the use of *nfkb1^−/−^* mice as an ideal model to dissociate the biology of pathological inflammaging from healthy chronological aging and to investigate therapeutic interventions in age-related chronic disease.

In this study, we report that modest exercise rebalances pre-established inflammatory and metabolic disturbances in aged *nfkb1^−/−^* mice, reverses hepato-steatosis, suppresses cellular senescence, and prevents liver tumorigenesis.

## Materials and Methods

### Mice for aged exercise studies

All experiments were performed on aged male *nfkb1^−/−^* on a pure C57BL/6 background between 16 and 19 mo of age. The *nfkb1^−/−^* were bred in-house as homozygous lines and housed in groups of four to six. Weekly weights were recorded from 64 wk of age. All animals were sacrificed at 19 mo of age, and all organs were harvested and either fixed in 4% paraformaldehyde or snap frozen and stored at −80°C. Fixed tissues were processed, embedded in paraffin, and cut at 4-μm thickness.

### Exercise intervention

Sixteen-month-old male *nfkb1^−/−^* mice were treadmill exercised (Panlab with air puff unit and SEDACOM Data Transfer Software V2.0) three times a week (Monday, Wednesday, and Friday) at the same time in the morning for 3 mo. The exercise routine comprised 5-min warmup at the speed of 14 cm/s, 20-min at 20 cm/s, and 5-min cool down at 14 cm/s. Sedentary animals were transported to the same room, weighed, and checked alongside the exercise mice, but were not treadmill exercised.

### Open field test

Activity was measured prior starting the exercise intervention (for both exercise and sedentary groups) and 1 and 2 mo after starting the intervention using a tablet with the MouseTrapp software, measuring steps taken, distance covered, and speed within 5 min of time. Mice were acclimatized to the testing room 1 h prior to behavioral monitoring and were not tested on the same day as exercise routine was performed.

### Histological assessment of liver disease

Aged *nfkb1^−/−^* liver sections were histologically examined by a liver pathologist (D.T.), severity of steatosis, hepatocyte ballooning, and inflammation were scored according to Ref. 20, and presence of tumors within the liver was assessed and scored.

### Statistical analysis

Linear regression analysis and ANOVA were used for correlation analysis using the SPSS package. Data are shown as means ± SEM. Excel was used to perform an unpaired *t* test in which *p < 0.05, **p < 0.01, or ***p < 0.001 was considered significant.

### Histological stains

Liver sections were formalin fixed, paraffin embedded, and stained with H&E and 0.1% Sirius Red Picric acid solution following standard procedures. Ki67 and reticulin stains were immunostained at the Department of Cellular Pathology, Royal Victoria Infirmary Hospital, Newcastle upon Tyne, U.K.

### Immunohistochemistry and immunofluorescence

Formalin-fixed, paraffin-embedded liver sections were used for staining. Endogenous peroxidase activity was blocked with hydrogen peroxide, and Ag retrieval was performed using 0.01% pronase for neutrophil elastase 1:200 (ab21595; Abcam), Ag unmasking solution for cleaved caspase-3 1:200 (Asp^175^) (5A1E) (9664; Cell Signaling Technology), 4HNE 1:50 (MHN-020P; JalCA), PCNA 1:7000 (ab18197; Abcam), CD4 1:100 (14-9766-82; eBioscience), B220 1:200 (ab64100; Abcam), CD68 1:200 (OABB00472; Aviva Systems Biology), NKp46/NCR1 1:50 (AF2225; R&D Systems), 1 mM EDTA (pH 9) for CD8 1:100 (14-0808-82; eBioscience), Foxp3 1:100 (14-5773-82; eBioscience), CD44var (v6) 1:200 (BMS145; eBioscience), phospho-Histone H2A.X 1:100 (Ser^139^) (20E3) (9718; Cell Signaling), and 1 mM EDTA (pH 8) for CD3 1:100 (MCA1477; Serotec). Tissue was blocked using Avidin/Biotin Blocking Kit (Vector Laboratories), followed by 20% swine serum in PBS and primary Ab incubation overnight at 4°C. The next day slides were washed and incubated with secondary Abs for 2 h (goat anti-rat (STAR 131B; Bio-Rad Laboratories), polyclonal swine anti-rabbit Igs/biotinylated (E0353; Dako), rabbit anti-goat (A21222; Life Technologies), and polyclonal rabbit anti-mouse Igs/biotinylated (E0354; Dako), all diluted 1:200, followed by Vectastain Elite ABC Reagent incubation for an hour. DAB peroxidase substrate kit was used to visualize Ags and slides were counterstained with Mayer’s hematoxylin. For 4HNE the mouse on mouse kit (Vector) was used.

Images were taken using the Nikon microscope and 15 high power (×20) fields were imaged and counted.

### RNA in situ hybridization for p21 detection

RNA in situ hybridization was performed after RNAscope protocol from Advanced Cell Diagnostics. Paraffin sections were deparaffinized with Histoclear, rehydrated in graded ethanol, and H_2_O_2_ was applied for 10 min at room temperature, followed by two washes in H_2_O. Sections were placed in hot retrieval reagent and heated for 15 min. After washes in H_2_O and 100% ethanol, sections were air dried. Sections were treated with protease plus for 30 min at 40°C, washed with H_2_O, and incubated with target probe (p21, no. 408551) for 2 h at 40°C. Afterward, slides were washed with H_2_O, followed by incubation with AMP1 (30 min at 40°C) and next washed with wash buffer (WB) and AMP2 (15 min at 40°C), WB and AMP3 (30 min at 40°C), WB and AMP4 (15 min at 40°C), WB and AMP5 (30 min at room temperature) and WB, and finally, AMP6 (15 min at room temperature). Finally, an RNAscope 2.5 HD Reagent kit-RED was used for chromogenic labeling. After counterstaining with hematoxylin, sections were mounted.

### TUNEL assay

Apoptotic cells were detected using the in situ cell death detection kit (11684817910; Roche) on formalin-fixed, paraffin-embedded liver sections and following the manufacturer’s protocol instructions.

### IDO enzyme assay

IDO activity was measured by HPLC as previously described (21). Briefly, previously snap-frozen liver tissue was homogenized in PBS at 100 mg of tissue per milliliter, lysed (2× freeze/thaw), and incubated with a substrate solution (100 mM potassium phosphate buffer [pH 6] containing 50 μM methylene blue, 50 mM ascorbate, 0.4 mM l-tryptophan, and 20 μg catalase; all from Sigma-Aldrich) for 0 or 2 h at 37°C. Reaction was stopped and protein precipitated with 1:10 perchloric acid 60% (Sigma-Aldrich); samples were centrifuged and filtered, and the kynurenine (KYN) levels were measured by HPLC. Results are expressed as picomoles of KYN (0–2 h/mg) of protein.

KYN and tryptophan present in the tissue homogenates were also analyzed by diluting sample with sodium acetate to a final concentration of 15 mM, then precipitated with 1:10 perchloric acid 60% (Sigma-Aldrich); samples were centrifuged and filtered, and the KYN levels were measured by HPLC. The same procedure was applied for kynurenic acid measurement, but a postcolumn mixer mixed the samples with a solution of zinc acetate 200 mM and ammonium acetate 500 mM before detection.

### Cytokine array

Mouse blood serum and mouse liver protein samples were sent to Canada to Eve Technologies for a Mouse Cytokine 31-Plex Discovery Assay screening. One-hundred-microliter volume liver protein with a concentration of 1000 μg was prepared according the standard protein extraction protocol. Mouse serum was diluted 2-fold in PBS.

### Lipidomics

Hepatic lipids were extracted using the Folch method (22). Chloroform/methanol (2:1, 1 ml) was added to 30 mg of liver tissue and homogenized using a TissueLyser (QIAGEN, Manchester, U.K.). Deionized water (400 μl) was added, and the samples were thoroughly mixed. The layers were separated by centrifuging (12,000 × *g*, 15 min). The organic layer was evaporated under a stream of nitrogen and reconstituted in chloroform/methanol (2:1, 600 μl) and diluted 1:50 in isopropanol/acetonitrile/water (2:1:1), also incorporating an internal standard (IS) mixture. For the serum lipidomics, 15 μl of serum was thoroughly mixed with 300 μl deionized water, 150 μl IS mixture in methanol, and 750 μl methyl tertiary-butyl ether in a glass vial. Following centrifugation, the upper layer was removed, dried down under nitrogen, and reconstituted in chloroform/methanol (1:1, 100 μl). This was then diluted 1:10 in isopropanol/acetonitrile/water (2:1:1).

The IS mixture was composed of isotopically labeled standards from Avanti Polar Lipids and included the following: *N*-palmitoyl-d31-d-*erythro*-sphingosine (C16-d31 ceramide), pentadecanoic-d29 acid (15:0-d29 free fatty acid [FFA]), heptadecanoic-d33 acid (17:0-d33 FFA), eicosanoic-d39 acid (20:0-d39 FFA), tetradecylphosphatidylcholine-d42 (14:0-d29 LPC-d13), 1-palmitoyl(d31)-2-oleyl-sn-glycero-3–phosphatidylcholine (PC; 16:0-d31-18:1 PC), 1-palmitoyl(d31)-2-oleyl-sn-glycero-3-phosphoethanolamine (16:0-d31-18:1 PE), 1-palmitoyl-d31-2-oleoyl-sn-glycero-3-[phospho-rac-(1-glycerol)] (16:0-d31-18:1 PG), *N*-palmitoyl(d31)-*d*-erythro-sphingosylphosphorylcholine (16:0-d31 SM), glyceryl tri(pentadecanoate-d29) (45:0-d87 total hepatic triglyceride [TAG]), and glyceryl tri(hexadecanoate-d31) (48:0-d93 TAG).

Samples were analyzed by liquid chromatography–mass spectrometry (MS) using an Accela HPLC system coupled to an LTQ Orbitrap Elite (Thermo Fisher Scientific, Hemel Hempstead, U.K.). Five microliters of sample was injected onto an Acuity C18 BEH column (50 × 2.1 mm, 1.7 μm; Waters, Warrington, U.K.) with a column temperature of 55°C. Mobile phase A was acetonitrile/water 60:40, and mobile phase B was isopropanol/acetonitrile 90:10, both of which included 10 mM ammonium formate (positive ion mode) or 10 mM ammonium acetate (negative ion mode). A gradient run was used at a flow rate of 0.5 ml/min, with starting conditions 40% B, increasing to 54, 81, and 99% B at 4.8, 5.8, and 8 min, respectively, holding at 99% B for 0.5 min before re-equilibrating with starting conditions for 1.5 min. The source was heated to 375°C, the desolvation temperature was 380°C, and desolvation gas flow was 40 arbitrary units. Spectra were acquired in positive and negative ion mode in the range of 200–1000 *m/z* at 60,000 mass resolution.

Raw data were converted to mzML format for processing using xcms (23). Lipid signals were normalized to the appropriate IS and to the weight of the tissue (or volume of serum). Lipid identification by accurate mass was achieved by searching against an in-house library of lipid *m/z* ratios computed for all combinations of common fatty acids, lipid head groups, and anticipated adducts. Tandem MS was used to confirm lipid class and identify acyl chain composition where possible.

### Lymphoid structure quantification

Liver slides were scanned, digitalized, and visualized on the ImageScope/Leica eSlides manager. Lymphoid structures were counted across all liver lobes and their size measured in (micrometers) diameter.

### Telomere immunofluorescence in situ hybridization

After γ-H2A.X immunofluorescence, slides were washed in PBS, cross-linked with 4% paraformaldehyde for 20 min, and dehydrated in graded ethanol. Sections were denatured for 10 min at 80°C in hybridization buffer (70% formamide [Sigma-Aldrich], 25 mM MgCl_2_, 0.1 M Tris [pH 7.2], and 5% blocking reagent [Roche Diagnostics] containing 25 μg/ml peptide nucleic acid probe [Applied Biosystems]), followed by hybridization for 2 h at room temperature in the dark. Slides were then washed in 70% formamide in 2 × SSC for 15 min and then washed in 2 × SSC for 10 min, followed by a 10-min PBS wash. Slides were then incubated with DAPI, mounted in VECTASHIELD, and imaged using *Z*-stacking (a minimum of 40 optical slices with ×63 objective; Leica). Whole-image stacks were used to count telomere-associated DNA damage response foci.

### RNA isolation and real-time PCR

RNA was isolated from mouse liver tissue previously snap frozen in liquid nitrogen using the RNeasy Mini Kit (QIAGEN). After RNA quantification and treatment with DNase, cDNA was synthesized using Promega random primers. SYBR Green quantitative real-time PCR was performed using the primers listed in was performed using the primers listed: Alox5 forward (F) 5′-ACTTTGTCGGCTATCTGGGAG-3′, reverse (R) 5′-CAGAAACAGGATGATCCGCTT-3′; CXCL10 F 5′-AAGTGCTGCCGTCATTTTCT-3′, R 5′-GTGGCAATGATCTCAAC-3′; GAPDH F 5′-GCACAGTCAAGGCCGAGAAT-3′, R 5′-GCCTTCTCCATGGTGGTGAA-3′; G6pC F 5′-GTCGTGGCTGGAGTCTTG-3′, R 5′-CGGAGGCTGGCATTGTAG-3′; 3-hydroxyanthranilic acid oxygenase (HAAO) F 5′-GGAGGCCCCAATACCAGGA-3′, R 5′-TATAGGCACGTCCCGGTGTT-3′; IDO1 F 5′-CAAAGCAATCCCCACTGTATCC-3′, R 5′-ACAAAGTCACGCATCCTCTTAAA-3′; IDO2 F 5′-CCTCATCCCTCCTTCCTTTC-3′, R 5′-GGAGCAATTGCCTGGTATGT-3′; LpCat2 F 5′-CGGCCCGCTTTGACATTTC-3′, R 5′-GCAACCTTTCCTTTCACGGTAAC-3′; PCK1 F 5′-GGTATTGAACTGACAGACTC-3′, R 5′-CCAGTTGTTGACCAAAGG-3′; quinolinic acid phosphoribosyltransferase (QPRT) F 5′-CCGGGCCTCAATTTTGCATC-3′, R 5′-GGTGTTAAGAGCCACCCGTT-3′; tryptophan 2,3-dioxygenase (TDO) 2 F 5′-AGGAACATGCTCAAGGTGATAGC-3′, R 5′-CTGTAGACTCTGGAAGCCTGAT-3′; TNF-α F 5′-CCCTCACACTCAGATCATCTT-3′, R 5′-GCTACGACGTGGGCTACAG-3′; KYN aminotransferase (KAT)1 F 5′-CGAAGGCTGGAAGGGATCG-3′, R 5′-GCGGTGAGAAGTCAGGGAA-3′; KYN 3-mono-oxygenase (KMO) F 5′-TGATGTGTACGAAGCTAGGGA-3′, R 5′-TCATGGGCACACCTTTGGAAA-3′; kynureninase (KYNU) F 5′-TCAAACCCTCCCATTTTGTTGG-3′, R 5′-CCCCTTGTTTTCGGTGTTATCTT-3′; and 18S F 5′-TAGAGGGACAAGTGGCGTTC-3′, R 5′-CGCTGAGCCAGTCAGTGT-3′.

### Isolation of whole-tissue lysates

Approximately 5 mg of liver tissue in radioimmunoprecipitation assay (RIPA) buffer containing protease and phosphatase inhibitors was mechanically disrupted with TissueLyser II (QIAGEN). Samples were placed on ice for 30 min and centrifuged at maximum speed for 15 min at 4°C. Supernatants were quantified using the detergent-compatible protein assay kit from Bio-Rad Laboratories.

### Western blots

Total liver tissue lysates were fractioned by 10% acrylamide gel. The proteins were then transferred to a nitrocellulose membrane and blocked in 5% milk protein diluted in TBST (0.1%) for 1 h. All primary Ab incubations were overnight: NF-κB p65 (8242s; Cell Signaling Technology), acetyl–NF-κB p65 (Lys^310^) (3045s; Cell Signaling Technology), β-actin (A5441; Sigma-Aldrich), acetyllysine (ab80178; Abcam), IKBα (4814S; Cell Signaling Technology), Sirt1 (07-131; MilliporeSigma), PPAR γ coactivator-1a1 (PGC-1α; NBP1-04676; Novus Biologicals), and PBEF (nicotinamide phosphoribosyltransferase [NAMPT]) (sc-393444; Santa Cruz Biotechnology). Next day, membranes were washed in TBST and incubated with secondary Abs (anti-mouse (A4416; Sigma-Aldrich) and anti-rabbit (7074S; Cell Signaling Technology) for 1 h. Proteins were detected with chemiluminescence (Amersham Bioscience).

### Immunoprecipitation

Liver tissue samples were prepared from liver tissues as previously stated, with the exception of the buffer used (immunoprecipitation [IP] RIPA buffer, 50 mM Tris-HCl [pH 7.4], 1% NP-40, 150 mM NaCl, 0.25% sodium deoxycholate, 5 mM EDTA, and protease and phosphatase inhibitors). One hundred micrograms of protein were added to 100 μl of IP RIPA buffer and incubated overnight at 4°C with 2 μl of the Ab of interest. The same amount of protein was taken as the input. Fifty microliters of Protein G–Sepharose (P3296; Sigma-Aldrich) were added to each sample, excluding the input ones, and incubated for further 2 h at 4°C. The complexes were washed three times with IP RIPA buffer and then resuspended in 2× Laemmli buffer and prepared for Western blot.

### ELISA for IL-6

IL-6 protein levels in tissue lysates were quantified using DuoSet ELISA Kit as per the manufacturer’s instructions (DY406; R&D Systems, Minneapolis, MN) and normalized to tissue protein concentration.

### NAD/NADH assay

NAD^+^ was measured in snap-frozen mouse liver tissue using the NAD/NADH-Glo Assay (Promega) following the manufacturer’s protocol description.

### Sirtuin activity

Sirtuin (SIRT) activity in snap-frozen mouse liver tissue was measured with the Sirtuin Activity Assay Kit (Fluorometric) from BioVision following the manufacturer’s protocol description.

## Results

### Modest aerobic exercise suppresses cellular senescence and tumor development

**FIGURE 1.**
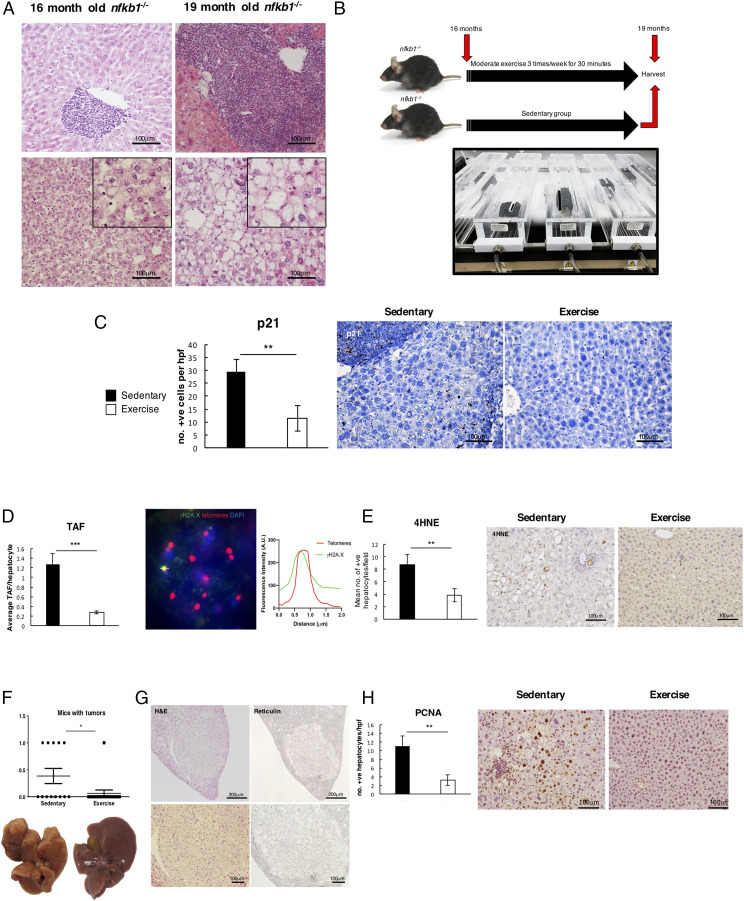
Exercise suppresses cell senescence and tumor development. (**A**) Representative pictures of livers of 16-mo (left)– and 19-mo (right)–aged *nfkb1^−/−^* mice. Images are H&E-stained liver sections showing initial stages of steatosis and inflammation in 16-mo-old *nfkb1^−/−^* mice and a more severe nonalcoholic steatohepatitis phenotype with increased inflammatory infiltrate at 19 mo of age. (**B**) Diagram depicting the exercise intervention and timeline. (**C**) Representative images of p21 staining with graph showing average number of positive p21–stained cells in livers of sedentary and exercised livers. (**D**) Representative images of TAF staining with average number of positive TAF counted per hepatocyte in sedentary and exercised livers. (**E**) Representative images of 4HNE stain and graph with average number of positive cells in livers of sedentary and exercised mice. (**F**) Graph shows number of mice with tumors in sedentary and exercised mice. (**G**) Representative images of H&E and reticulin stains in the livers of the sedentary mice. (**H**) Representative images of PCNA staining in the livers of sedentary and exercised mice with graphs showing average number of positive cells in both groups. Data are means ± SEM. Data of one study (sedentary, *n* = 13; exercised, *n* = 16). Statistical significance was determined using an unpaired two-tailed Student *t* test. *p < 0.05, **p < 0.01, ***p < 0.001 compared with control.

We have previously described how the inflammaging phenotype of *nfkb1^−/−^* mice leads to development of chronic liver disease and premature aging, resulting in early mortality at around 20 mo (10, 14). Histological examination of the livers of *nfkb1^−/−^* mice at an earlier age of 16 mo revealed the presence of inflammation and steatosis, which became progressively more severe by 19 mo (Fig. 1A). We asked if administering an intervention of structured modest aerobic exercise between 16 and 19 mo would suppress liver cell aging and prevent this disease progression. To this end, mice were aged to 16 mo and separated into sedentary and exercise groups. The sedentary animals were limited to normal activity within the constraints of their cage, whereas the exercise group were treadmill exercised 30 min/d, three times a week at a speed of 8–12 m/min without an incline for a total of 3 mo (Fig. 1B). This aerobic treadmill regimen with a 5% incline was previously deemed as moderate based on 65–70% of maximal oxygen uptake (24). To determine the effects of exercise on cellular aging, we carried out immunohistochemistry on liver sections for the senescence marker p21 (Fig. 1C). Exercise significantly reduced numbers of p21-positive hepatocytes compared with sedentary groups. We also detected fewer numbers of hepatocytes carrying damaged telomeres (Fig. 1D); this was assessed by fluorescence in situ hybridization for telomere-associated DNA damage foci (TAF), which is an established quantifiable marker for stable telomere DNA damage and senescence (3, 10). Lower levels of 4HNE staining were indicative of exercise protecting from reactive oxygen species–induced lipid peroxidation, which underlies DNA damage in this model (Fig. 1E). Roughly 40% of aged *nfkb1^−/−^* mice spontaneously develop a liver tumor by 20 mo (14). In the current study, 38.5% of the sedentary group developed tumors, as determined by visual inspection and microscopic analysis of H&E- and reticulin-stained liver sections (Fig. 1F, 1G). Remarkably, only one animal in the exercise group had evidence of tumor growth. This antitumorigenic effect was accompanied by reduced numbers of PCNA^+^ and yH2AX^+^ hepatocytes (Fig. 1H, Supplemental Fig. 1). Collectively, these data suggest that modest exercise applied as intervention in the context of pre-established liver disease will suppress inflammation-driven cellular aging and tumor development.

### Exercise reduces liver inflammation

**FIGURE 2.**
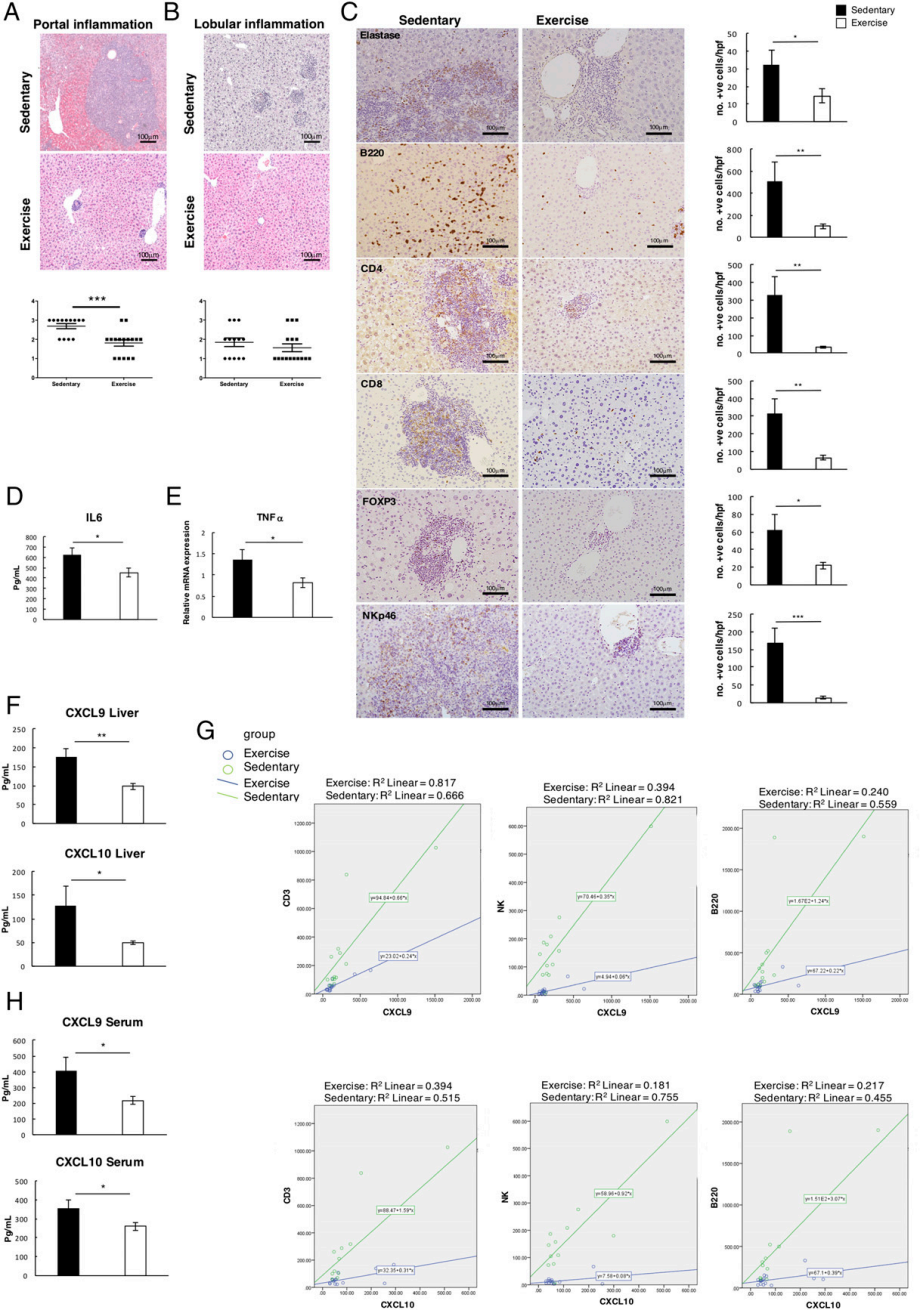
Exercise reduces liver inflammation. (**A** and **B**) Representative H&E images of portal and lobular inflammation in the livers of sedentary and exercised mice. The graphs below show portal and lobular inflammation scores, portal inflammation score indicated as 0, none; 1, mild, at least one portal tract with mild inflammation; 2, moderate, at least one portal tract with moderate inflammation; and 3, severe, at least one portal tract with severe inflammation. Lobular inflammation score indicated as 0, none; 1, mild, up to one necroinflammatory focus; 2, moderate, at least two to four necroinflammatory focus; and 3, severe, more than five necroinflammatory focus. (**C**) Representative immunohistochemistry images of neutrophil elastase, B220, CD4, CD8, FOXP3, and NKp46 in livers of sedentary mice and graphs with mean numbers of quantified positive-stained cells. (**D** and **E**) Graphs showing IL-6 at protein level and TNFa at the mRNA level in livers of sedentary and exercised mice. (**F**) Graph showing CXCL9 and CXCL10 in liver protein of sedentary and exercised mice and (**G**) linear models correlating CXCL9 and CXCL10 against CD3, B220, and NK cells (group variables: CXCL9/CD3, *p* = 0.009; CXCL10/CD3, *p* = 0.008; CXCL9/B220, *p* = 0.027; CXCL10/B220, *p* = 0.015; CXCL9/NK, *p* = 0.0001; and CXCL10/NK, *p* = 0.0001). (**H**) Graph showing CXCL9 and CXCL10 in blood serum of sedentary and exercised mice at 19 mo. Data of one study (sedentary, *n* = 13; exercised, *n* = 16). Data are means ± SEM. Statistical significance was determined using an unpaired two-tailed Student *t* test. *p < 0.05, **p < 0.01, ***p < 0.001 compared with control.

We were next interested to determine if exercise impacts on the underlying chronic inflammatory phenotype. The majority of sedentary *nfkb1^−/−^* mice progressed to severe portal inflammation as well as mild to moderate lobular inflammation at 19 mo (Fig. 2A, 2B). Lobular inflammation was mixed and predominantly lymphocytic with fewer neutrophils present. There were no foci of pure acute inflammation. Lobular inflammation scoring was applied according to Kleiner et al. (20) by assessing the presence of necroinflammatory foci (spotty necrosis) in the lobules at ×10 magnification. Inflammatory infiltrates were significantly reduced in the exercise cohort and this was particularly striking in the portal regions. Lobular inflammation measured by numbers of lobular necroinflammatory foci was scored as predominantly mild in the exercise group (up to one necroinflammatory foci) and mild to moderate (up to four foci) in the sedentary group (Fig. 2B). The anti-inflammatory effects of exercise were noted for multiple immune cell types, including neutrophils (elastase), B cells (B220), NK cells (NKp4.6), and CD4^+^, and CD8^+^ and Foxp3^+^ T cells in the exercise group (Fig. 2C). Notably, there was no observed change in the number of CD68^+^ macrophages (Supplemental Fig. 2A). We also observed significant decreases in levels of hepatic IL-6 and TNF-α in the exercise group (Fig. 2D, 2E). Liver protein levels of CXCL9 and -10, which are chemotactic proteins for T, B, and NK cells, were also significantly reduced by exercise, and these data correlated with the observed reduction in recruitment of T, B, and NK cells (Fig. 2F, 2G). In addition, we also detected a similar lymphocytic infiltrate in the lung and the stomach of aged *nfkb1^−/−^* mice, which was also significantly reduced by exercise (Supplemental Fig. 2B). Moreover, protein levels of CXCL9 and CXCL10 were expressed at lower levels in the serum of exercised mice, indicating a systemic impact of the intervention and reduced levels of CXCL10 in the lung that correlated with a reduction in infiltrating T and B cells (Fig. 2H, Supplemental Fig. 2C, 2D).

### Suppression of ectopic lymphoid structures and growth of tumor progenitor cells

**FIGURE 3.**
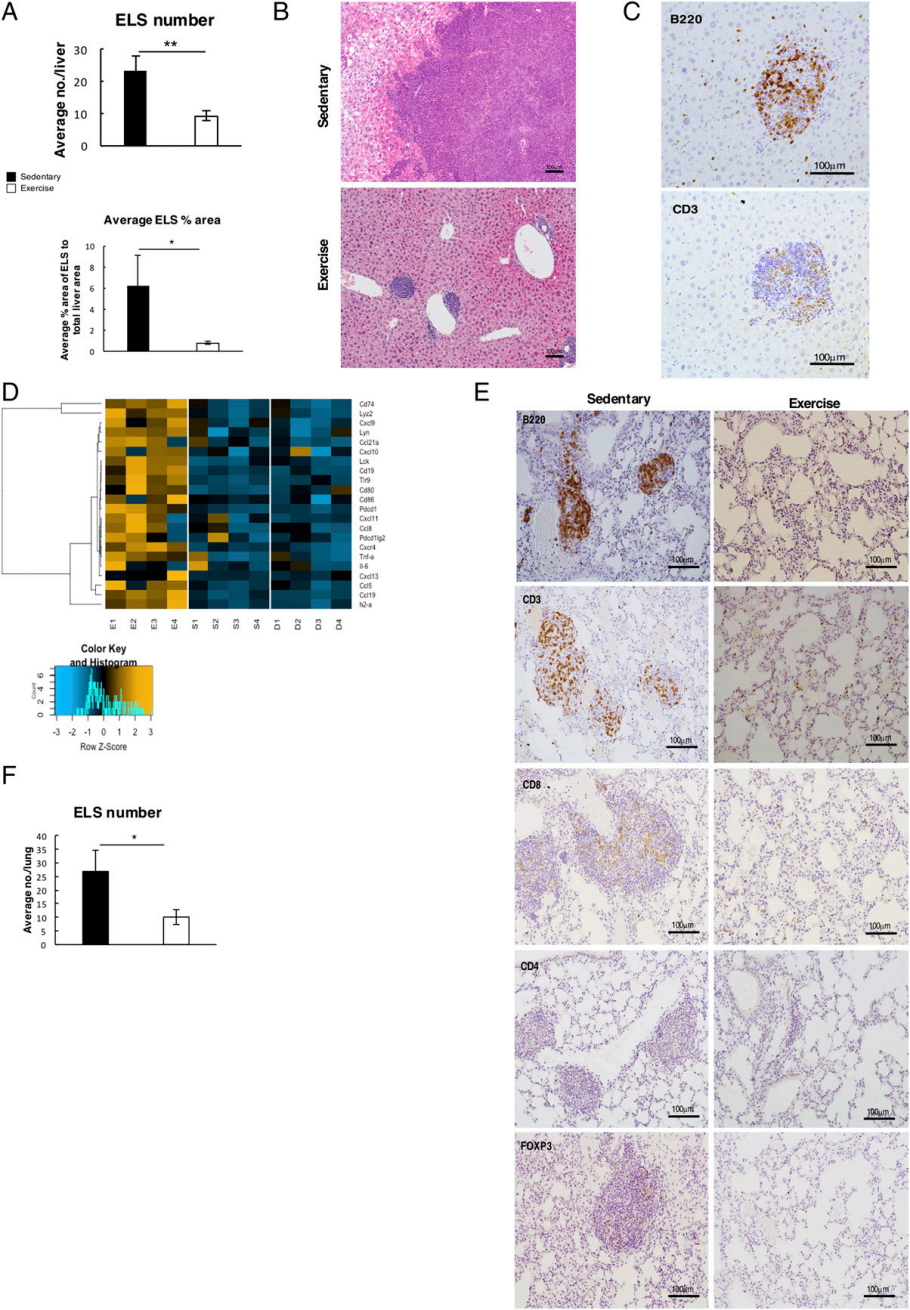
Lymphocytic aggregates resembling ELS are reduced with exercise. (**A**) Top graphs shows average number of ELS counted in the livers of sedentary (*n* = 13) and exercised (*n* = 16) mice. Bottom graph shows average measurement of size of ELS in each liver of sedentary and exercised mice and is represented as percentage of total liver area. (**B**) Representative H&E images of ELS in livers of sedentary and exercised mice. (**C**) Representative images of ELS in 19-mo-old nfkb1^−/−^ mice stained by B220 and CD3 showing ELS are mainly composed of T and B cells. (**D**) Image showing representative NanoString analysis of laser capture microdissected ELS (E1–4) compared with area surrounding the ELS (S1–4) and distinct liver tissue with no visible ELS (D1–4). (**E**) Representative images of ELS-like structures and immune cell infiltrate in the lungs of sedentary and exercise mice. (**F**) Graph shows average number of ELS counted in the lungs of sedentary and exercised mice. Data of one study (sedentary, *n* = 13; exercised, *n* = 16). Data are means ± SEM. Statistical significance was determined using an unpaired two-tailed Student *t* test. *p < 0.05, **p < 0.01 compared with control.

A striking observation from histological examination of the liver and lungs of aged *nfkb1^−/−^* mice was what appeared to be large aggregates of T and B lymphocytes (Fig. 3A–F). These structures appeared to resemble ectopic lymphoid structures (ELS), which in humans, are associated with a variety of chronic inflammatory conditions as well as with inflammation-driven cancers (25–27). Indeed, liver ELS may function as microniches for tumor development (26). To characterize the immune aggregates in the liver, we used laser capture microdissection to isolate RNA from suspected ELS, peri-ELS, and non-ELS tissue and then performed NanoString to determine gene expression. This analysis revealed a distinct immune-rich gene expression profile that confirmed the immune aggregates as ELS; indeed, the profile contained a similar immune signature to that previously used to assess ELS in human tissues (28). We also detected an enrichment of immune effector genes involved in T, B, and NK cell function and immune cell recruitment (Fig. 3D). We were also interested to determine if the ELS contained any tumor progenitor cells that can be detected by immunohistochemistry for the established cancer stem cell marker CD44v6. As shown in (Supplemental Fig. 2E), this analysis revealed the presence of CD44v6 tumor progenitors within larger ELS of sedentary aged mice. Of note, CD44v6-positive cells were surrounded by immune cells staining positively for the lipid peroxidation marker 4HNE in the sedentary mice (Supplemental Fig. 2F). Hence, ELS in this model appear to be microniches that may support tumor initiation from an inflammatory environment. In addition, we could not detect CD44v6 progenitors in liver ELS of exercised animals, indicating that the tumorigenic potential of these structures was diminished by the intervention (Fig. 3E). As the ELS in the exercised mice were so small, it was not possible to conclude differences in immune cell proliferation or apoptosis using stains for Ki67, PCNA, and TUNEL when compared with the huge structures detected in the sedentary group (Supplemental Fig. 2G–I). Hence, we are unable to attribute alterations in immune cell growth or survival as an explanation for the impact of exercise on ELS; instead, we speculate that reduced expression of immune mediators, such as CXCL9 and CXCL10, required for ELS assembly may explain the smaller size and fewer numbers of immune cell aggregates.

### Resolution of liver steatosis and modulation of lipid composition

**FIGURE 4.**
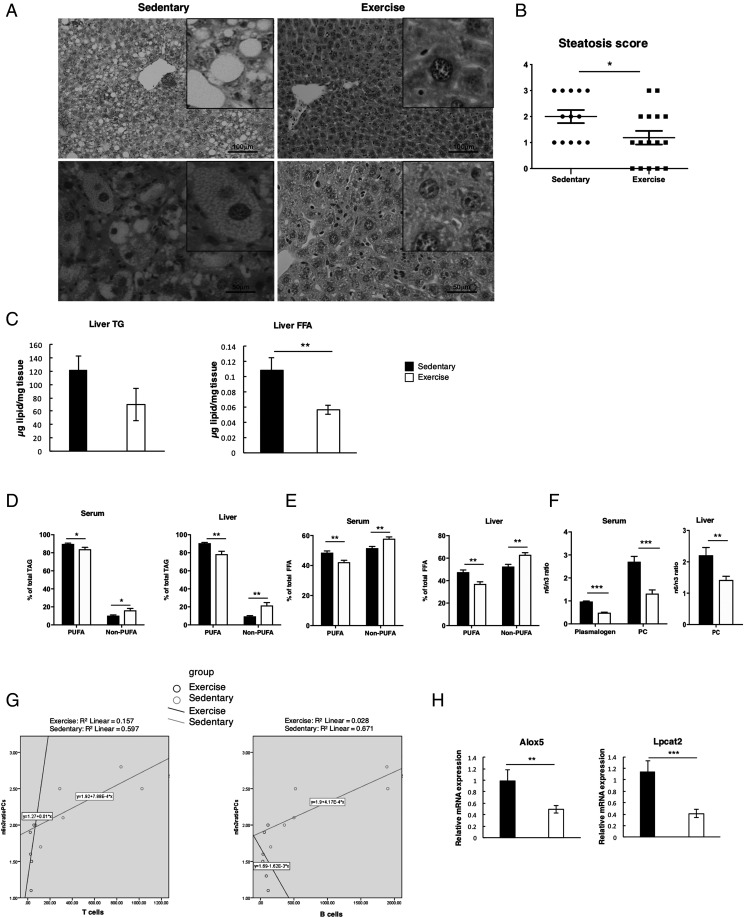
Moderate aerobic exercise improves liver steatosis and changes lipid composition. (**A**) Representative H&E images of livers of 19-mo-old sedentary and exercised *nfkb1^−/−^* mice. (**B**) Graph shows steatosis score indicated as 0, none; 1, mild; 2, moderate; and 3, severe in sedentary (*n* = 13) and exercise (*n* = 16) mice. (**C**) Graphs depicting liver triglycerides (TG) and liver FFA in sedentary and exercised mice. (**D**) Graphs depicting percentage of total TAG in serum and liver of sedentary and exercised mice. (**E**) Graphs depicting percentage of total FFA in serum and liver. (**F**) Graphs depicting n6/n3 ratio in liver PC and in serum plasmalogen and PC. (**G**) SPSS correlations: Left graph: In the sedentary group (green line), a higher number of CD3 cells positively correlates with a higher n6/n3 ratio of PCs. Right graph: The sedentary group (green line) shows a higher n6/n3 ratio correlates with a higher number of B220^+^, B cells. No correlation between n6/n3 with CD3 or B220 is found in the exercise group (group variables: n6/n3 ratio of PCs/CD3, *p* = 0.226; and n6/n3 ratio of PCs/B220, *p* = 0.230), (**H**) Relative mRNA levels of genes Alox5 and Lpcat2 in liver of sedentary and exercised groups. Data of one study (sedentary, *n* = 13; exercised, *n* = 16). Data are means ± SEM. Statistical significance was determined using an unpaired two-tailed Student *t* test. *p < 0.05, **p < 0.01, ***p < 0.001 compared with control.

H&E-stained sections confirmed that steatosis was established in all sedentary *nfkb1^−/−^* mice at 19 mo of age, with 38% having progressed to a severe grade 3 steatosis in all three zones of the liver (Fig. 4A). Steatosis extent was graded according to Kleiner et al. (20) at ×4 magnification (grades 0–3: 0, <5%; 1, 5% to <1/3; 2, 1/3–2/3; and 3, >2/3 of hepatocytes affected). In sedentary mice, steatosis was of mixed type (macrovesicular and microvesicular) microvesicular steatosis, as noted in higher magnification (×10), predominatedly affecting patches of more than 10 hepatocytes. Steatosis grade was significantly reduced with exercise, with no evidence of microvesicular steatosis and 31% of mice lacking steatosis in any of the lobes or zones of the liver (Fig. 4A, 4B). This histological observation was supported by biochemistry, with TAG content and total FFA being decreased in the exercise group (Fig. 4C). To more closely investigate changes in lipid composition, we performed hepatic and serum lipid profiling using liquid chromatography–MS. This lipidomic analysis revealed that exercise promoted an altered fatty acyl chain composition. Within the TAG class, there was a significant relative reduction in species containing one or more polyunsaturated fatty acid (PUFA), and a concomitant relative increase in non-PUFA containing TAG species in both liver and serum from the exercised mice (Fig. 4D). A similar trend was observed for hepatic and serum FFA, with a shift toward non-PUFA species in the exercised group (Fig. 4E). This trend was not found in phospholipids, with significant increases in both PUFA and non-PUFA containing PC noted after exercise. However, there was a dramatic shift in the ratio of PC-containing *n-6* fatty acids (e.g., arachidonic acid [AA], 20:4) and *n-3* fatty acids (e.g., docosahexanoic acid, 22:6). Exercise exerted a significant decrease in the hepatic PC *n-6*/*n-3* ratio and a significant decrease in the serum *n-6/n-3* ratio of PCs and plasmalogens (Fig. 4F). The balance between *n-6* and *n-3* fatty acids is considered an important driver of the proinflammatory shift from bland steatosis to steatohepatitis (29). Because *n-6* and *n-3* PUFA tend to be precursors to pro- and anti-inflammatory lipid mediators, respectively, a decrease in the *n-6/n-3* ratio suggests an amelioration of the inflammatory phenotype of *nfkb1^−/−^* mice in the exercise group. Supporting this, is the strong correlation between infiltrating CD3^+^ T cells and B220^+^ B cells with the hepatic PC *n-6/n-3* ratio in sedentary, but not in the exercised, mice (Fig. 4G).

Of the lipid mediators, most of those derived from AA tend to be proinflammatory. We therefore investigated hepatic gene expression for AA-related metabolic pathways, including the synthesis of eicosanoids via the lipoxygenase pathway (*Alox5*), and the reincorporation of AA into the membrane (*Lpcat2*). These genes displayed significantly reduced hepatic expression in the exercise group (Fig. 4H). Taken together, the data suggest that exercise reduces age-related inflammation, both through a change in hepatic cellular composition and through changes to the hepatic and systemic lipid signature. It is also plausible that the observed changes in lipid composition reflect the dramatic reduction in infiltrating and proliferating immune cells in exercised mice.

### Exercise protects from age-related decline in activity and body condition

We next asked if the metabolic and inflammatory changes occurring as a consequence of exercise have general health benefits beyond the prevention of liver disease and tumor development. We have reported that premature aging of *nfkb1^−/−^* mice is associated with decreased motor and cognitive function and a decline in overall body condition (10). To determine if exercise would have beneficial effects on activity and condition of these mice, open field tests were carried out at 16, 17, and 18 mo (Supplemental Fig. 3A). In these tests we measured speed of movement, distance traveled, and numbers of steps (touches) taken over a fixed period as measures of activity. These measures are useful indicators of general wellbeing because activity is known to decline in aging humans and particularly in aged individuals with a chronic liver disease (30). Accordingly, our analysis of aged *nfkb1^−/−^* mice revealed a steady decline in activity levels over the 3-mo period of investigation (Supplemental Fig. 3B). Strikingly, in the exercise group there was no evidence of activity decline over the same 3-mo period. In addition, overall body condition, as evaluated by weekly body condition scoring, was maintained in the exercise group in contrast to its gradual decline in the sedentary group (Supplemental Fig. 3C). However, we observed no significant change in overall bodyweight, liver/bodyweight ratio or muscle mass at 19 mo (Supplemental Fig. 3D, 3E). We conclude that even when applied in the context of a pre-established age-associated chronic inflammatory disease, a regimen of regular but modest aerobic exercise prevents the decline of general health and promotes wellbeing.

### Exercise increases hepatic SIRT activity and deacetylation of NF-κB and PGC-1α

**FIGURE 5.**
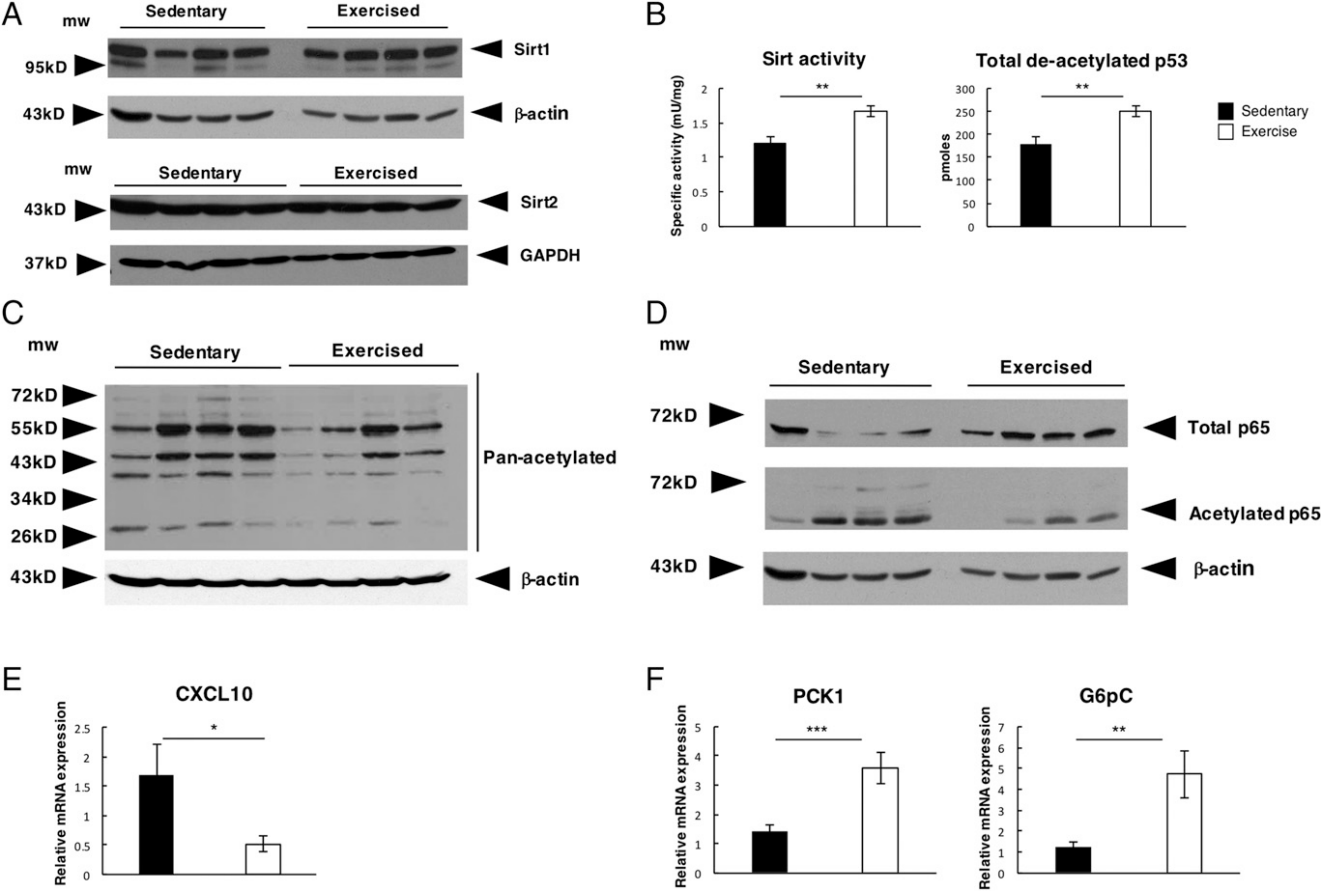
Exercise increases SIRT activity and deacetylation of NF-κB and PGC-1α. (**A**)Western blot analysis of hepatic Sirt1 and Sirt2 protein levels (**B**) SIRT activity levels in livers of sedentary and exercised mice and total de-acetylated p53 peptide levels. (**C**) Western blot of pan-acetyl lysine in livers of sedentary and exercised mice with housekeeping control β-actin. (**D**) Western blots of total p65 and acetylated p65 negative for the housekeeping control β-actin. (**E**) Graphs showing relative mRNA expression of CXCL10 in liver of sedentary (*n* = 13) and exercised (*n* = 16) mice. (**F**) Graphs depicting relative mRNA expression of PGC1α regulated genes PCK1 and G6pC in liver of sedentary and exercised mice. Data are means ± SEM. Data of one study (sedentary, *n* = 13; exercised, *n* = 16). Statistical significance was determined using an unpaired two-tailed Student *t* test. *p < 0.05, **p < 0.01, ***p < 0.001 compared with control.

Having demonstrated that exercise impacts on both inflammation and metabolism, we hypothesized that these changes may reflect alterations in the expression of SIRT1 and SIRT2, which are nuclear deacetylases capable of modulating the transcriptional control of inflammatory and metabolic pathways (31, 32). However, both hepatic SIRT1 and SIRT2 showed no differences in expression between sedentary and exercised animals (Fig. 5A). We therefore measured SIRT deacetylase activity and found this to be significantly increased in the livers of the exercised group, demonstrated by an increase in levels of deacetylated p53 (Fig. 5B). We also observed an overall reduction in total hepatic protein acetylation in exercised mice, implying broader control over target proteins (Fig. 5C). The NF-κB subunit p65 is a central mediator of inflammation and is tightly regulated by a number of posttranslational modifications, including deacetylation by SIRT, known to suppress NF-κB activation and inflammatory gene expression (31, 33). Although there was an overall increase in total levels of p65 in the exercise group compared with sedentary mice, we confirmed an overall decrease in hepatic p65 acetylation in the livers of the exercised animals (Fig. 5D). In conjunction with lowered NF-κB activity, we also observed a significant decrease in the expression of NF-κB–regulated inflammatory genes, including TNF-α and CXCL10 (Figs. 2D, 5E)

SIRT1 is a major regulator of hepatic lipid and glucose homeostasis, this function operating at least in part through deacetylation of the transcriptional coactivator PGC-1α (34–36). PGC-1α is a major adaptor protein required for maintaining metabolic homeostasis and is strongly activated under conditions of energy limitation including exercise (35). In response to SIRT1 control, deacetylated PGC-1α boosts glucose output by binding to HNF-4a and FOX01 and increases the expression of key enzymes involved in glucose homeostasis including Pck1 and G6Pc (37). Although we did not observe any difference in the protein level of hepatic PGC-1α, we did observe an overall reduction in PGC-1α acetylation in the exercised mice group and a corresponding increase in expression of the PGC-1α–regulated genes Pck1 and G6Pc confirmatory of the broad downstream consequences of modulating SIRT activity (Fig. 5F, Supplemental Fig. 4A).

We conclude that moderate exercise promotes reprogramming of dysregulated inflammatory and metabolic pathways arising from parainflammation.

### Exercise boosts tryptophan metabolism and NAD^+^ levels in the aged liver

**FIGURE 6.**
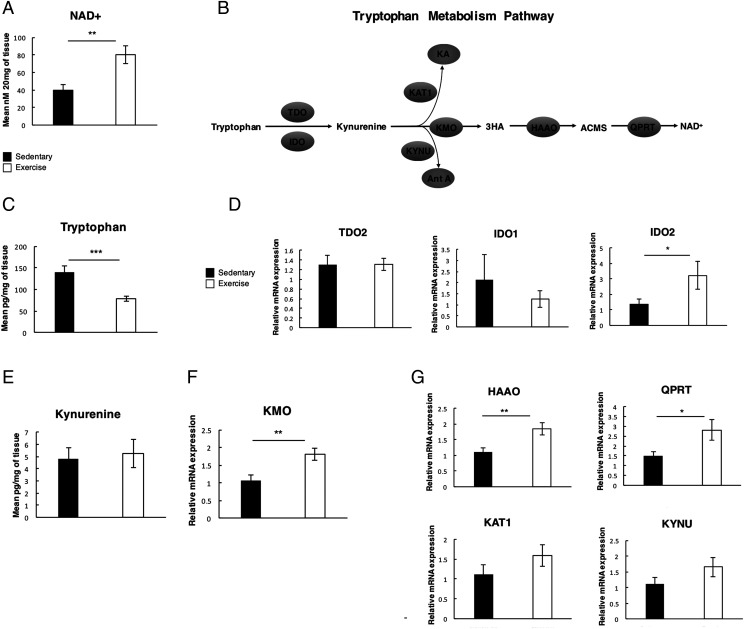
Modest exercise boosts tryptophan metabolism and NAD^+^ levels in the aged liver. (**A**) Graph depicting mean NAD^+^ levels measured in the livers of sedentary and exercised *nfkb1^−/−^* mice. (**B**) Simplified diagram of the de novo tryptophan metabolism pathway (**C**) Graph depicting mean tryptophan levels measured in the livers of sedentary and exercised *nfkb1^−/−^* mice (sedentary, *n* = 13; exercised, *n* = 16). (**D**) Graphs depicting relative mRNA expression of TDO2, IDO1, and IDO2 in the liver. (**E**) Graph depicting mean KYN levels measured in the livers of sedentary and exercised *nfkb1^−/−^* mice. (**F**) Graph depicting relative mRNA expression of KMO in livers of sedentary and exercised *nfkb1^−/−^* mice. (**G**) Graph depicting relative mRNA expression of genes involved in the tryptophan metabolism pathway: KYNU, KAT1, HAAO, and QPRT in livers of sedentary and exercised *nfkb1^−/−^* mice. Data of one study (sedentary, *n* = 13; exercised, *n* = 16). Data are means ± SEM. Statistical significance was determined using an unpaired two-tailed Student *t* test. *p < 0.05, **p < 0.01, ***p < 0.001 compared with control.

SIRT are dependent on oxidized NAD (NAD^+^) as a substrate for their deacetylase function (38–42). NAD^+^ is generated via a de novo synthetic pathway that predominantly occurs in the liver or it can be recycled from nicotinamide via a salvage pathway reliant on the rate-limiting enzyme NAMPT (43). Elevating NAD^+^ levels has been reported to have major therapeutic implications for metabolism and aging (41). We therefore asked if the anti-inflammatory and improved metabolic profile of exercised *nfkb1^−/−^* mice is associated with altered NAD^+^ levels. Indeed, measurement of hepatic NAD^+^ revealed a significant increase in the exercise group (Fig. 6A). To determine the mechanism underlying this metabolic change, we first examined the de novo synthetic pathway that begins with the conversion of tryptophan to KYN by TDO or IDO. (Fig. 6B). Tryptophan levels were significantly reduced in the livers from the exercise group (Fig. 6C) despite food intake being consistent between groups (Supplemental Fig. 4B). Hepatic TDO and IDO1 expression was unchanged by exercise; however, levels of IDO2 transcript were upregulated in the exercised group (Fig. 6D). Of note, we did not detect differences in hepatic levels KYN between exercised and sedentary mice (Fig. 6E), implying its rapid metabolism through the downstream KYN pathway. This is supported by enhanced expression of KMO in exercised mice (Fig. 6F). KMO competes with KAT to generate 3-hydroxykynurenine, which is subsequently metabolized by KYNU, HAAO, and finally QPRT to generate NAD^+^ (Fig. 6B). In addition to upregulation of KMO, we also recorded significantly increased expression of transcripts for HAAO and QPRT with exercise, whereas levels of KAT and KYNU were unchanged (Fig. 6G). Hence, exercise upregulates the expression of at least four key metabolic enzymes (IDO2, KMO, HAAO, and QPRT) in the hepatic NAD^+^ synthetic pathway. By contrast, we did not observe changes in NAMPT levels that might have supported enhanced NAD^+^ salvage (Supplemental Fig. 4C).

## Discussion

Epidemiological and interventional studies indicate that exercise as a component of a medical care plan can improve quality of life and may extend life in the context of chronic disease; however, mechanisms are ill-defined (44). In this study, we report that interventional exercise alone, even at a modest intensity, is sufficient to ameliorate pre-established and progressively worsening inflammatory and metabolic pathologies that actively promote unhealthy aging and cancer. We observed exercise to stimulate a reduction in telomere DNA damage and cellular senescence, which are classic hallmarks of aging. Additionally, exercise promoted reversion of hepatic steatosis, resolution of inflammation, and prevention of tumor development.

A number of distinct mechanisms are proposed to explain the anti-inflammatory benefits of exercise in humans (45). High-intensity or long-duration exercise is associated with an acute spike in IL-6, which subsequently leads to induction of anti-inflammatory IL-10 and IL-1RA (46). IL-10 can also be generated from exercise-induced mobilization of regulatory T cells (47). Prolonged strenuous exercise is associated with decreased expression of TLRs on monocytes and reduced stimulation of proinflammatory molecules by LPS (48). Exercise studies in mice and humans indicate downregulation of ICAM1 as a potential mechanism for reducing inflammatory cell recruitment and immune cell interactions (49). Where exercise reduces visceral fat, a reduction in proinflammatory adipokines can contribute to systemic inflammation (50). A further documented mechanism is stimulation of the sensory nervous system leading to release of adrenocorticotropic hormone and subsequent stimulation of anti-inflammatory cortisol (51). A caveat with the majority of these studies is that their conclusions are based on measurements of inflammatory regulators in peripheral blood, which do not necessarily reflect the inflammatory state of tissues. Furthermore, age and level of pre-existing systemic or tissue-specific inflammation are not controlled across these different human studies. It should also be recognized that mechanistic differences will arise based on the nature of the performed exercise (e.g., aerobic versus resistance training) as well as its intensity, duration, and frequency. As an example, rises in circulating IL-6 and anti-inflammatory IL-10 are not observed following repeated modest exercise despite observed health benefits (52). Further characterization of the ectopic lymphoid-like structures in the liver, including spatial assessment of follicular dendritic cells, follicular Th cells, lymphotoxin, and monoclonal B cells, where they form, and what happens in response to exercise is an essential next step. Data informing us of the composition and role of these structures (e.g., providing a tumor niche or developing local antitumor immune responses) at different stages of disease will help provide us with potential targeting at the correct point in time.

We propose that the *nfkb1^−/−^* mouse provides a unique experimental test bed for investigating the molecular mechanisms by which exercise positively impacts on the progression of age-related inflammatory processes within tissues.

Mechanistically, our data indicate that exercise stimulates the hepatic de novo NAD^+^ synthetic pathway and, in turn, enhanced activities of NAD^+^-dependent SIRT. Hepatic levels of NAD^+^ and SIRT1 (the best characterized member of the SIRT family), decline with aging and are accompanied by a simultaneous increase in accumulation of DNA damage (53). Conversely, experimental overexpression of SIRT1 lowers DNA damage and p16 levels, indicating its ability to suppress cellular senescence (54). We reveal that at least four key enzymes in the NAD^+^ synthetic pathway are induced by exercise training, this correlating with deacetylation of the SIRT1 targets p53, RelA, and PGC-1α, and decreased numbers of DNA-damaged hepatocytes.

Acetylated RelA plays a cardinal role in the control of inflammation and senescence (via activation of the senescence-associated secretory phenotype) (31, 55, 56). Hence, enhanced SIRT-dependent deacetylation of RelA provides a plausible explanation for exercise-induced suppression of liver inflammation. The liver is the major site for synthesis of NAD^+^ from tryptophan and is a source of the nucleotide for other organs (57), explaining not only the prevention of liver disease of exercised mice but also the observed improvements in lung and stomach pathologies. Elevation of hepatic NAD^+^ was accompanied by reductions in levels of triglyceride and FFA and a shift toward a more anti-inflammatory lipid signature within the liver. SIRT1 uses multiple mechanisms to modulate lipogenesis, including deacetylation of PGC-1α, to enhance PPARα-induced fatty acid β-oxidation and suppression of prolipogenic transcription factors SREBP-1 and ChREBP (58). SIRT1 also indirectly influences lipid metabolism through its modification of SIRT3 and SIRT6, both of which stimulate fatty acid β-oxidation (59, 60). The ability of SIRT1 to prevent cellular senescence via deacetylation of p53 provides an additional potential mechanism through which exercise may suppress fat accumulation in hepatocytes (61). Ogrodnik et al. (4) recently reported that senescence promotes fat accumulation in hepatocytes because of impaired mitochondrial function and demonstrated that genetic or pharmacological elimination of senescent hepatocytes prevents steatosis. NAD^+^ induction of SIRT1 by exercise therefore stimulates combined metabolic and inflammatory reprogramming in the liver responsible for the observed reversion of senescence and nonalcoholic fatty liver disease in exercised *nfkb1^−/−^* mice.

Although the therapeutic activation of SIRT has many potential benefits, the wide range of downstream targets, including basal transcriptional machinery, transcription factors, and coregulators, the complication of off-target effects are highly likely and will differ vastly between individuals (62). A different therapeutic approach would be to direct a more personalized targeting regimen and explore the therapeutic potential of individual downstream SIRT targets. For instance, therapeutic PGC-1α inhibitors to restore glucose homeostasis in type 2 diabetes or starve tumor cells in cancer (63, 64). However, other SIRT targets, such as NF-κB, are central regulators of inflammation, stress response, and cellular metabolism, etc. Approaches aimed at targeting upstream regulators of the NF-κB pathway have failed because of off-target effects. Therefore, we hypothesize that moderate exercise in aging individuals promotes a balanced activation of SIRT that restores normal metabolic and immune homeostasis as opposed to dysregulated immune cell activation, cellular survival, and proliferation. Further experimentation using scaled omics approaches will ultimately lead to a more detailed landscape of how moderate exercise improves healthy aging and uncover targetable biomarkers that define its mechanistic actions.

In summary, we have shown in a genetic model of inflammaging and progressive chronic disease that a regimen of structured mild aerobic exercise, which could reasonably be administered as an intervention in the elderly, reprograms disturbed metabolic and inflammatory processes. This results in the effective prevention of the establishment of tumorigenic microenvironments and age-related pathologies.

## Supplementary Material

Data Supplement
