# Supplementary material for: Moderate Exercise Inhibits Age-Related Inflammation, Liver Steatosis, Senescence, and Tumorigenesis
Source: J Immunol. 2021 Jan 13;206(4):904–16. doi: 10.4049/jimmunol.2001022 (PMC7851741; doi:10.4049/jimmunol.2001022)
Supplement: Data Supplement [file JI_2001022.zip › JI_2001022_Supplemental_Figures_1.pdf]

a

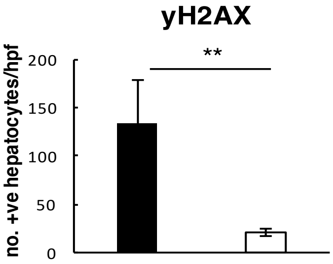

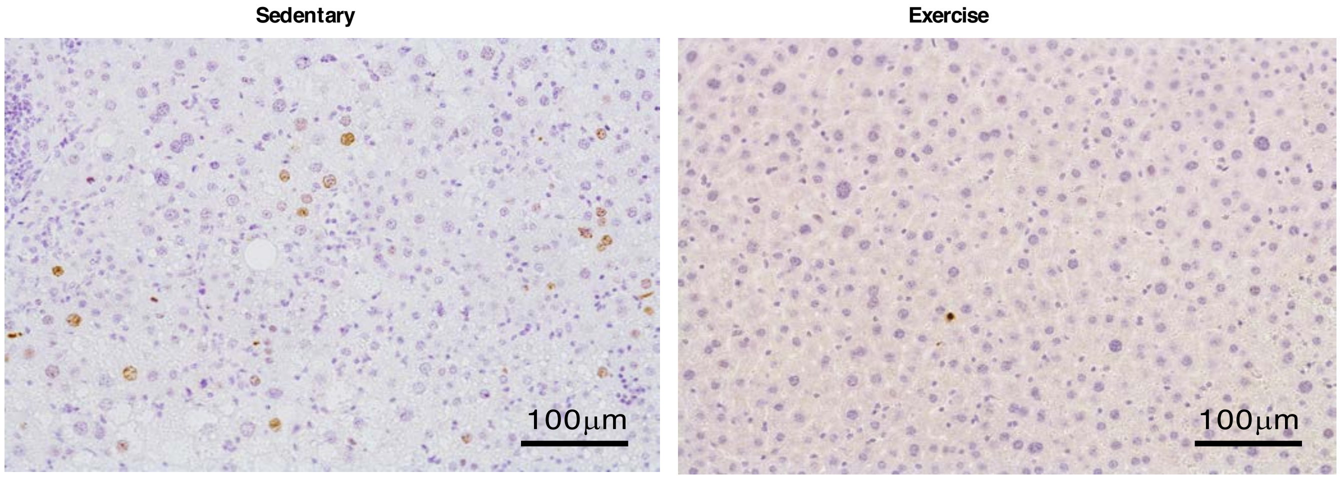

Supplemental Figure 1. Representative images of yH2AX stain in livers of sedentary and exercised mice with graph showing average number of positive cells.

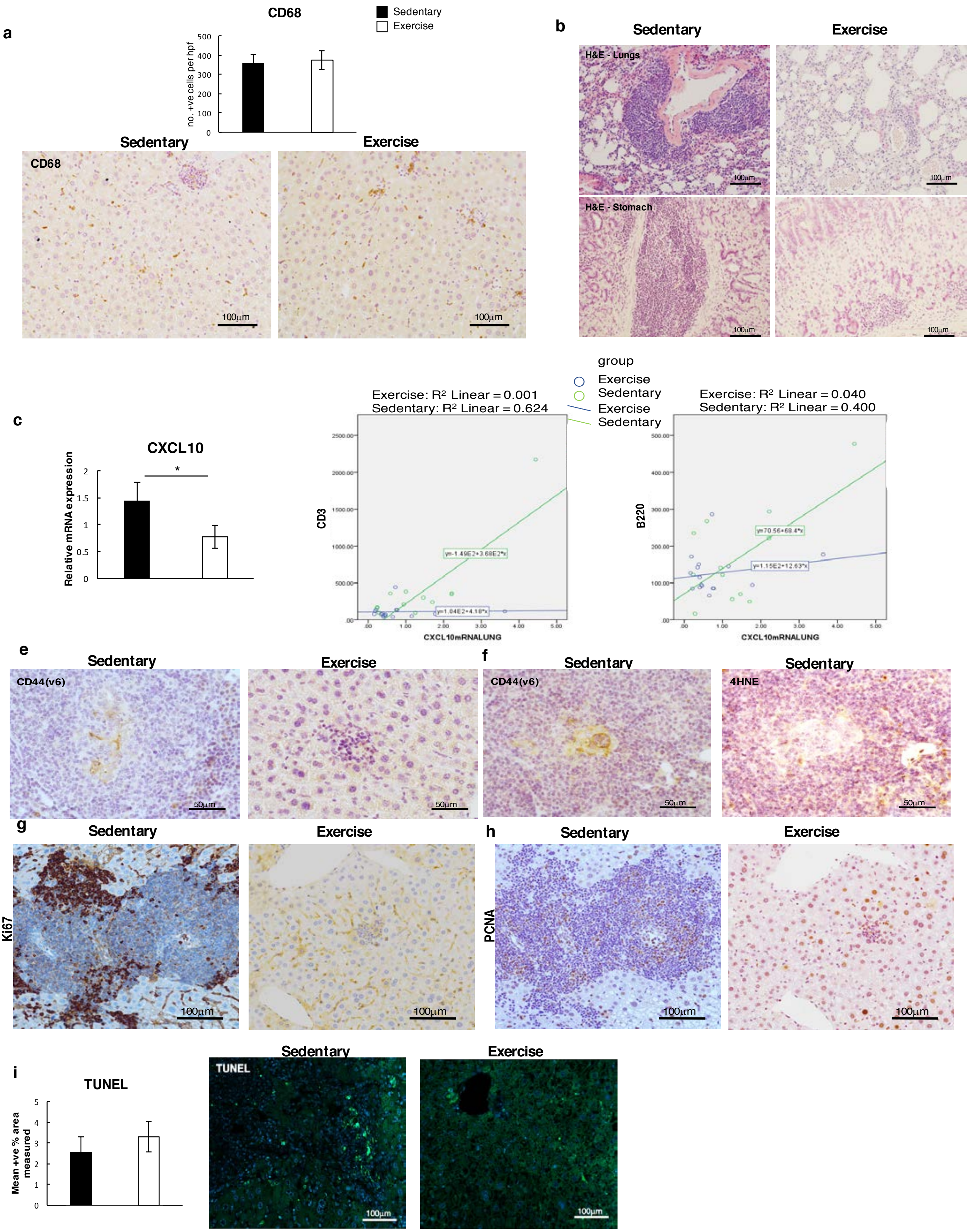

Supplemental Figure 2. (a) Representative images of CD68 stain in livers of sedentary and exercised mice with graph showing average number of positive cells. (b) Representative images of H&E staining in lungs and stomach of sedentary and exercised mice. (c) Relative mRNA expression of CXCL10 in lung cDNA of sedentary and exercised mice. (d) SPSS correlation between CD3 and B220 with mRNA levels of CXCL10 in the lungs of sedentary and exercised mice. (e) Representative images of CD44(v6) staining in ELS in livers of sedentary and exercised mice showing only positive staining in ELS found in sedentary mice. (f) Representative images of CD44(v6) and 4HNE staining in the same ELS of liver of sedentary mice showing positive lipid peroxidation around CD44(v6) positive cells. (g) Representative immunohistochemistry images of Ki67 and (h) PCNA in livers of sedentary and exercised mice showing cell proliferation in both groups scattered throughout ELS. (i) Representative images of TUNEL staining in livers of sedentary and exercised mice and mean positive staining within ELS was measured using ImageJ. Data are means ± s.e.m. Statistical significance was determined using an unpaired two-tailed Student *t*-test. *P<0.05, **P<0.01 or ***P<0.001 compared with control.

a

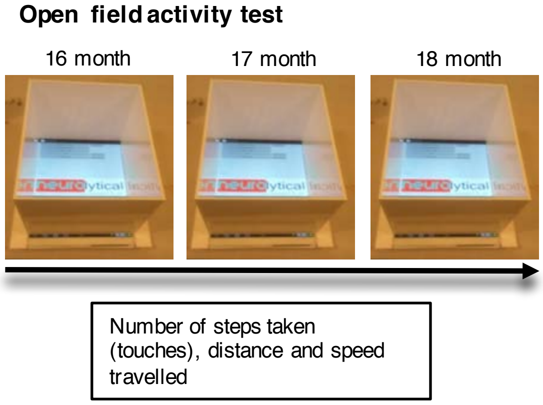

b

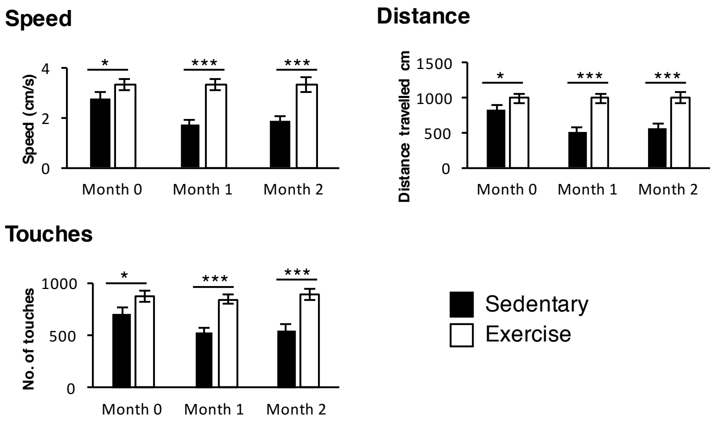

c

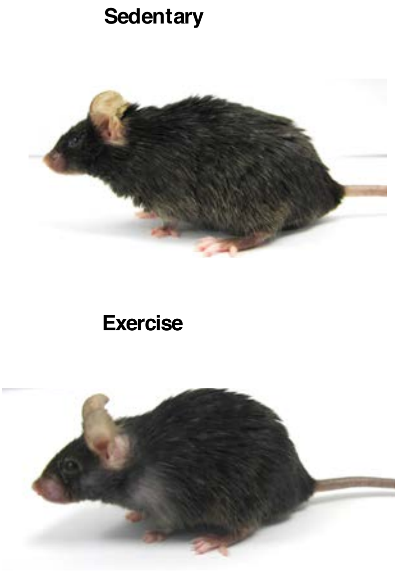

d

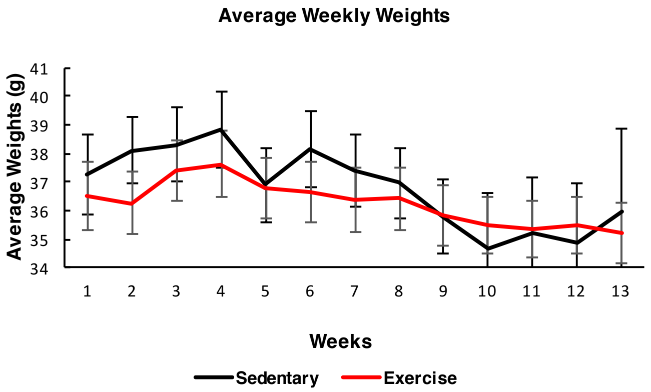

e

Average organ body weight ratio %

Organs:	Sedentary	Exercise	p values
Brain	1.33%	1.31%	0.43785854
Liver	4.94%	4.79%	0.351940159
Spleen	0.67%	0.45%	0.035824768
Heart	0.63%	0.66%	0.260780584
Lungs	0.66%	0.64%	0.376811125
Right Kidney	0.78%	0.81%	0.308645722
Left Kidney	0.79%	0.84%	0.227839286
Pancreas	0.47%	0.69%	0.031508725
Muscle	0.96%	1.03%	0.166789989
Fat	1.31%	1.10%	0.262976155

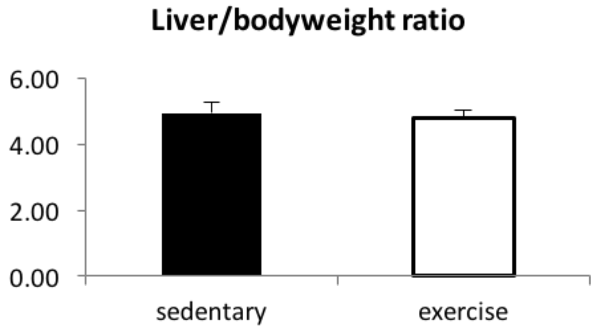

Supplemental Figure 3. Exercise maintains activity levels and body condition. (a) Image of open field activity test performed on both sedentary and exercised mice at 16, 17 and 18 months of age. Number of steps taken (recorded as touches on the tablet), distance and speed travelled were recorded through the Mousetrapp software. (b) Graphs show open field activity test results of sedentary and exercised mice at baseline levels (prior start of exercise routine) and one and two months into the exercise intervention study. (c) Representative images of the aged *nfkb1*$^{-/-}$ sedentary and exercised mice. (d) Graph showing average weekly weights recorded for each mouse over the course of the exercise intervention (sedentary n=13, exercised n=16). (e) Table showing average organ body weights ratio in % at harvest.Graph highlighting liver/bodyweight. Data are means ± s.e.m. Statistical significance was determined using an unpaired two-tailed Student *t*-test. *P<0.05, **P<0.01 or ***P<0.001 compared with control.

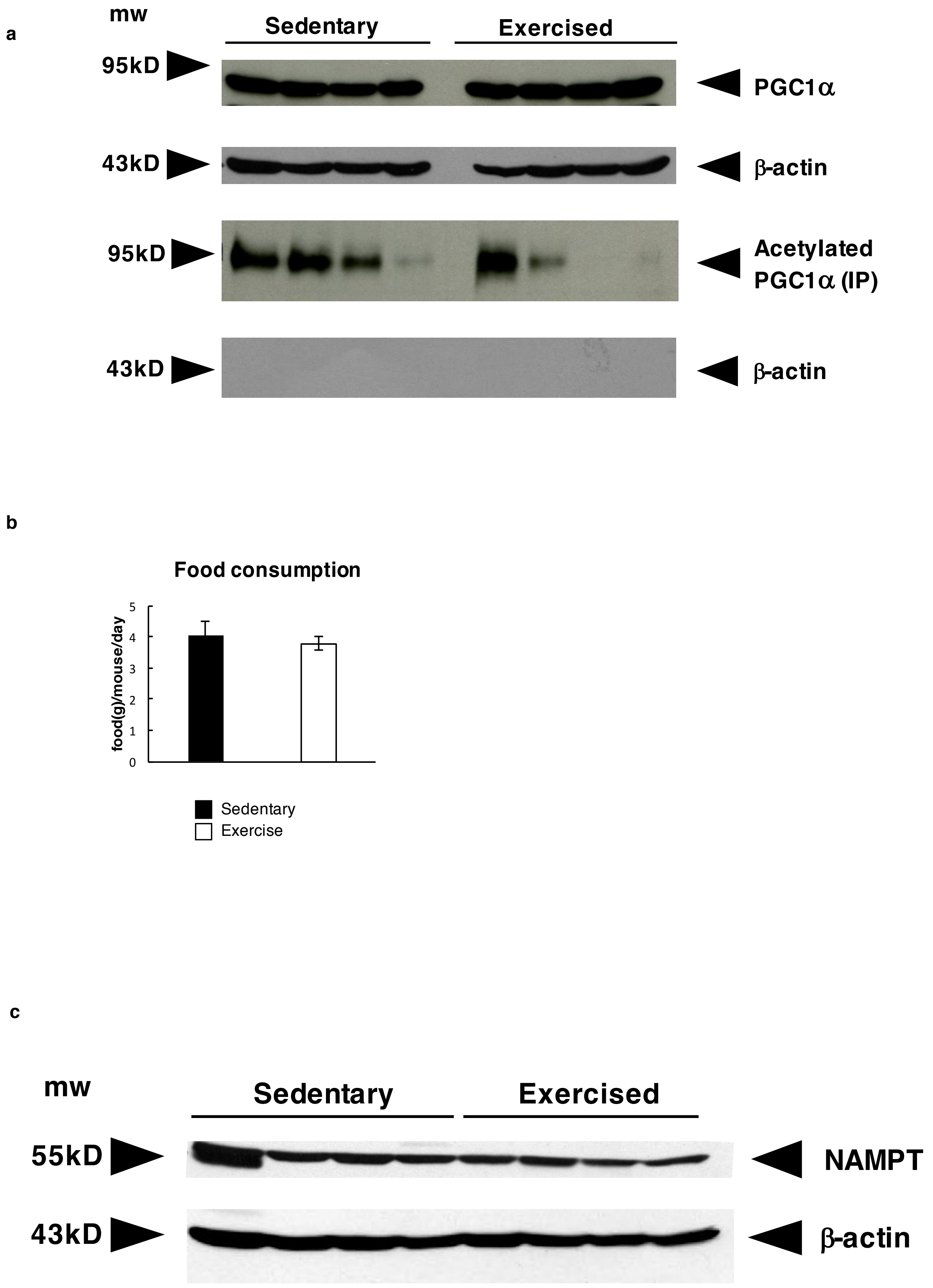

Supplemental Figure 4. (a) Western blot showing immunoprecipitation (IP) for pan-acetyl lysine and western blot for PGC1α, top band shows western blot for PGC1α in the input lysate used for IP n=4/group. (b) Graph showing food consumption of sedentary and exercised mice calculated per mouse per day. (c) Western blot of NAMPT in livers of sedentary and exercised mice with house-keeping control β-actin. Data are means ± s.e.m. Statistical significance was determined using an unpaired two-tailed Student *t*-test. *P<0.05, **P<0.01 or ***P<0.001 compared with control.
